# Supplementary material for: The Efficacy of an N-Acetylcysteine–Antibiotic Combination Therapy on Achromobacter xylosoxidans in a Cystic Fibrosis Sputum/Lung Cell Model
Source: Biomedicines. 2022 Nov 10;10(11):2886. doi: 10.3390/biomedicines10112886 (PMC9687303; doi:10.3390/biomedicines10112886)
Supplement: Supplementary file 1 [file biomedicines-10-02886-s001.zip › biomedicines-1886143-supplementary.pdf]

Supplementary Materials

The efficacy of an N-acetylcysteine -antibiotic combination therapy on *Achromobacter xylosoxidans* in a cystic fibrosis sputum/lung cell model.

Aditi Aiyer [1], Theerthankar Das [1,†], Gregory S. Whiteley [2,3], Trevor Glasbey [4], Frederik H. Kriel [4], Jessica Farrell [1,2], and Jim Manos [1,†]

1 Charles Perkins Centre, Infection, Immunity and Inflammation, School of Medical Sciences, The University of Sydney, Sydney, NSW 2006, Australia;
2 Whiteley Corporation, Level 5, 12 Mount Street North Sydney, Sydney, NSW 2060, Australia
3 School of Medicine, Western Sydney University, Sydney, NSW 2566, Australia
4 Whiteley Corporation, 19-23 Laverick Avenue, Tomago, NSW 2322, Australia
* Correspondence: aditi.aiyer@sydney.edu.au and das.ashishkumar@sydney.edu.au
† These authors contribute equally to this work.

The following figures and tables are included as Supplementary materials and are referred to in the manuscript titled above

1. *Assessment of changes in pH of media formulations*

Changes in pH were determined in the media preparations of DMEM and 20% ASMDM-1 were investigated for each $NAC_{neutral}$ antioxidant concentration outlined in **Table S1**. Readings were performed in triplicate using a pH meter (Mettler-Toledo, GmbH, Greifensee, Switzerland) that was calibrated using pH standards 4.0, 7.0 and 10.0. **Table S1** shows the pH values of the above media formulations at different concentrations of $NAC_{neutral}$.

Table S1. pH at different concentrations of $NAC_{neutral}$ in both DMEM and 20% ASMDM-1.

**Concentration (mg/mL)**	**pH (DMEM 100%)**			**pH (20% ASMDM-1 / 80% DMEM)**		
	Sample 1	*Sample 2*	*Sample 3*	*Sample 1*	*Sample 2*	*Sample 3*
1.025	7.87	7.86	7.88	7.62	7.63	7.62
2.05	7.69	7.72	7.75	7.65	7.64	7.64
4.1	7.73	7.74	7.75	7.64	7.61	7.63
8.2	7.56	7.57	7.58	7.59	7.60	7.59
16.3	7.24	7.36	7.38	7.59	7.58	7.59

2. *Optimal tolerance of bronchial epithelial cells (BEAS-2B) in ASMDM-1*

The optimal concentration of ASMDM-1 in BEAS-2B was determined using previously detailed methods with modifications. Briefly, BEAS-2B were seeded in 24-well plates (Corning Corp, New York, N.Y., USA) at 5 x 10^4 cells/mL. The plates were incubated at 37 ^{o}C (5% CO_2) until confluent. Following this, media was discarded, and cells were washed with 1 x Phosphate buffer saline (PBS: 137 mM NaCl, 2.7 mM KCl and 10 mM phosphate, pH 7.41) (POCD Healthcare, Sydney, Australia). DMEM or diluted ASMDM-1 was added to the cells at varying concentrations; 0-, 20-, 40-, 60- and 80% or 100% ASMDM-1. The plates were then placed into the IncuCyte Zoom® Live cell imaging system (Essen BioScience, United States) at 37°C and 5% (v/v) CO_2 for 24 h. Images of the BEAS-2B cells were captured at start (0 h) and then at 4 h intervals until the end of the experiment (24 h), using the standard inbuilt imaging system.washed with 1 x PBS.

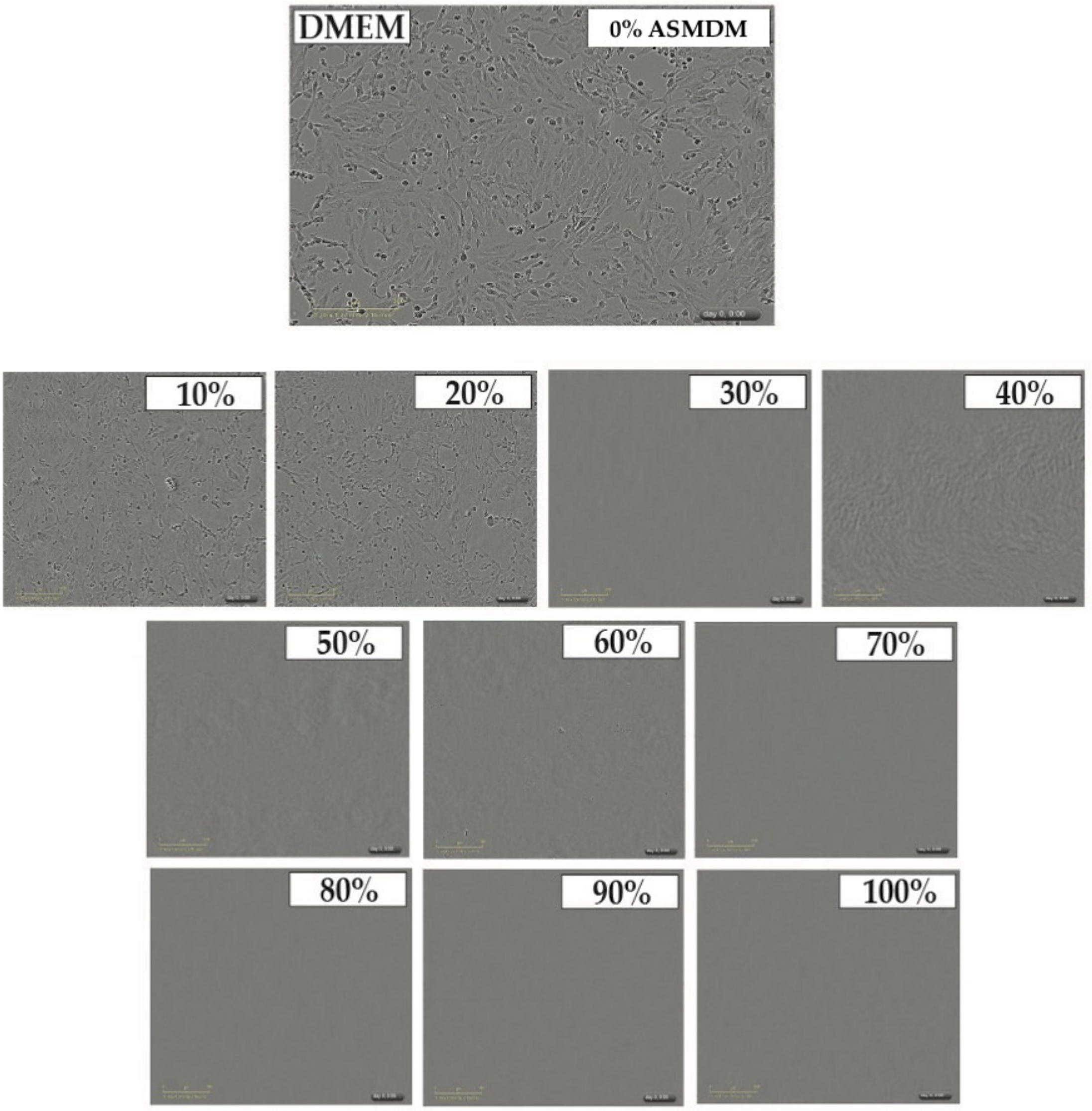

Figure S1. Images of BEAS-2B + varying concentrations of ASMDM-1 submerged culture. BEAS-2B cells were grown to confluence and imaged using the IncuCyte Zoom® Live cell Imaging and Analysis system. One snapshot was taken of each well to display the imaging capacity of each concentration of ASMDM-1 in DMEM. Imaging capacity is severely reduced after 20% ASMDM-1 condition as cells were not visible after this point.

3. *Optimal tolerance of* A. xylosoxidans *in ASMDM-1*

The optimal concentration of ASMDM-1 tolerated by *A. xylosoxidans* bacteria was determined as above using preivously detailed methods [1]. In brief, 10 µL of diluted bacterial cultures were added to wells containing ASMDM-1 suspended in DMEM at varying concentrations; 0-, 10-, 20-, 30-, 40- and 50%. Plates were then incubated at 37 °C for 24 h. Following this, a CFU count was established using the Whitley Automatic Spiral Plater (WASP) (Don Whitley Scientific, West Yorkshire, UK) where the WASP automatically plated 50 µL of diluted supernatant. TSA plates were incubated for 48 h at 37 °C following which colonies were enumerated and expressed as CFU/mL.

A. xylosoxidans *bacterial cell viability does not significantly change with increasing concentrations of ASMDM-1*

We determined *A. xylosoxidans* viability in varying concentrations of ASMDM-1 in comparison to DMEM alone (**Figure S2**). A maximum of 50% ASMDM-1 (diluted in 50% DMEM) was used. *A. xylosoxidans* was able to grow in all ASMDM concentrations and except for 40% ASMDM-1 in **Figure S2** ($p \leq 0.05$), there were no significant changes in bacterial growth in different ASMDM-1 concentrations relative to the control ($p > 0.05$).

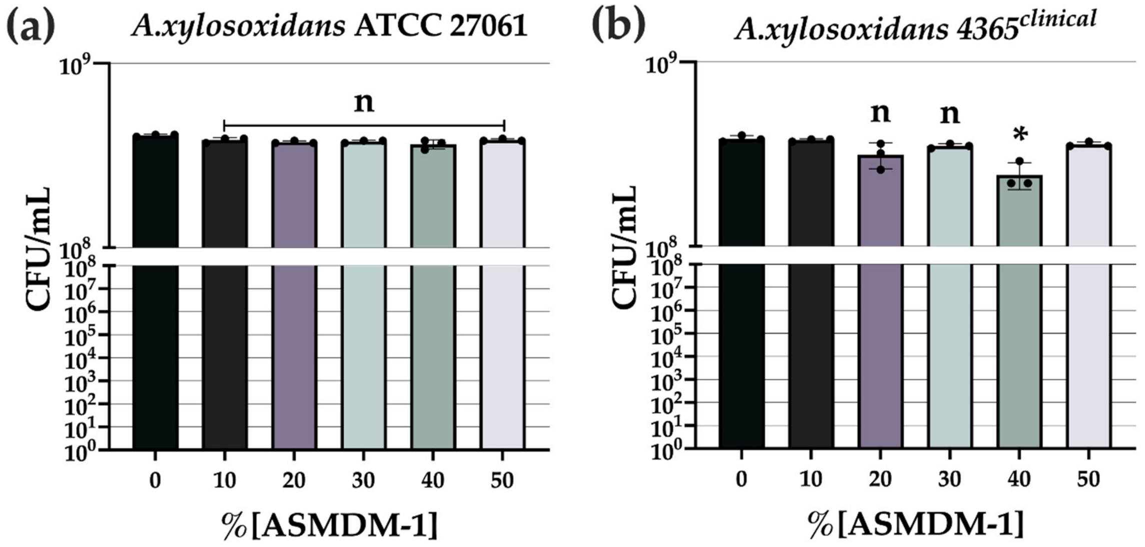

Figure S2. Assessment of ASMDM-1 effect on *A. xylosoxidans* bacterial growth. Diluted bacterial cultures of *A. xylosoxidans* (**a**) ATCC 27061 and (**b**) AX 4365 were inoculated in 24 well plates containing above concentrations of ASMDM-1 diluted in DMEM and incubated for 24 h. Bacterial loads were enumerated using CFU/mL. Statistical analyses were performed using unpaired Student's t-tests (with Welch's correction) and multiple comparison tests (Kruskal–Wallis test with Dunn's correction). Significance cut-offs were p > 0.05 (n), p ≤ 0.05 (*). Data represent an average of n = 3 biological replicates.

4. *BEAS-2B wound healing assay to measure epithelial repair capacity*

The wound healing assay has been used to assess the cell migration following artificial gap creation on a confluent BEAS-2B monolayer, as previously described [2]. In brief, BEAS-2B cells were seeded in 24-well plates at 5 x 10^4 cells/mL and then incubated at 37 ^{o}C until confluence achieved. Media was removed and the cells were washed with 1 x PBS, the confluent cell monolayer was scratched using a P200 pipette tip and washed again to remove any extra cell debris. The media is then replaced with DMEM or 20% ASMDM-1 medium. The plates were then placed into the IncuCyte Zoom® Live cell imaging system (Essen BioScience, United States) at 37°C and 5% (v/v) CO_2 for 24 h. Images of the BEAS-2B cells were captured at start (0 h) and then at 4 h intervals until the end of the experiment (24 h), using the standard inbuilt imaging system. After exporting images, wound areas were measured using an ImageJ wound healing plugin [2]. Briefly, a polygon selection tool was used to indicate wound area, and quantified the area (μm) of the wound. Wound healing rate (μm/hr) was determined using previously detailed methods [3]. Briefly, area was plotted against time and the aboslute value of the gradient of the linearly-fitted data and dividing it by 2 times the vertical length of the wound.

Would healing is not significantly different in 20% ASMDM-1 versus DMEM alone

To assess the effect of 20% ASMDM-1 versus DMEM alone on viability of cells, sheet migration was measured using wound healing (**Figure S3**). Wound healing rates were determined using equation detailed in Jonkman et al. (2014) [3] by taking the gradient of a graph plotted in μm^2 against time and dividing it by two times the length of the wound; yielding a μm/hr rate. The wound healing rates, a surrogate for epithelial health following tissue injury, between DMEM and 20% ASMDM-1 were not significantly different to each other ($p \leq 0.05$).

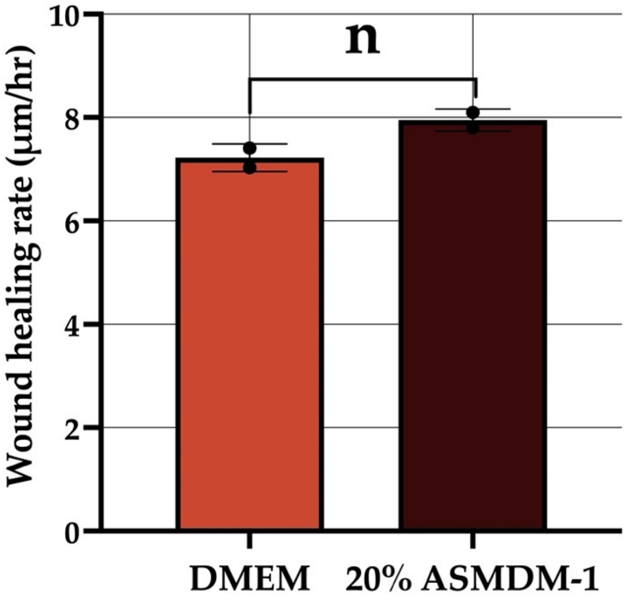

Figure S3. Wound healing not significantly different in DMEM alone versus 20% ASMDM-1. Wound closure of BEAS-2B cells was assessed using a scratch assay. Briefly, cells were grown to confluence in 24 well plates prior to being washed and scratched with a P200 pipette tip. Wound closure rates (μm/hr) were determined by measuring the gap area against time and plotting the slope against the vertical length of the wound. Statistical analyses were performed using unpaired Student's t-tests (with Welch's correction) and multiple comparison tests (Kruskal–Wallis test with Dunn's correction). Significance cut-offs were $p > 0.05$ (n), $p \leq 0.05$ (*). Data represent an average of n = 3 biological replicates.

References:

1. Wijers, C.D.; Vagedes, R.; Weingart, C. A novel method for investigating Burkholderia cenocepacia infections in patients with cystic fibrosis and other chronic diseases of the airways. *BMC Microbiol* **2016**, *16*, 200, doi:10.1186/s12866-016-0811-7.
2. Suarez-Arnedo, A.; Torres Figueroa, F.; Clavijo, C.; Arbeláez, P.; Cruz, J.C.; Muñoz-Camargo, C. An image J plugin for the high throughput image analysis of in vitro scratch wound healing assays. *PLoS One* **2020**, *15*, e0232565, doi:10.1371/journal.pone.0232565.
3. Jonkman, J.E.; Cathcart, J.A.; Xu, F.; Bartolini, M.E.; Amon, J.E.; Stevens, K.M.; Colarusso, P. An introduction to the wound healing assay using live-cell microscopy. *Cell Adh Migr* **2014**, *8*, 440-451, doi:10.4161/cam.36224.
